# Enhancement of cerebrovascular 4D flow MRI velocity fields using machine learning and computational fluid dynamics simulation data

**DOI:** 10.1038/s41598-021-89636-z

**Published:** 2021-05-13

**Authors:** David R. Rutkowski, Alejandro Roldán-Alzate, Kevin M. Johnson

**Affiliations:** 1 Mechanical Engineering, University of Wisconsin, Madison, WI USA; 2 Radiology, University of Wisconsin, 1111 Highland Ave, Madison, WI USA; 3 Medical Physics, University of Wisconsin, 1111 Highland Ave, Madison, WI USA

**Keywords:** Medical research, Engineering, Mathematics and computing, Fluid dynamics, Physics, Imaging techniques, Machine learning

## Abstract

Blood flow metrics obtained with four-dimensional (4D) flow phase contrast (PC) magnetic resonance imaging (MRI) can be of great value in clinical and experimental cerebrovascular analysis. However, limitations in both quantitative and qualitative analyses can result from errors inherent to PC MRI. One method that excels in creating low-error, physics-based, velocity fields is computational fluid dynamics (CFD). Augmentation of cerebral 4D flow MRI data with CFD-informed neural networks may provide a method to produce highly accurate physiological flow fields. In this preliminary study, the potential utility of such a method was demonstrated by using high resolution patient-specific CFD data to train a convolutional neural network, and then using the trained network to enhance MRI-derived velocity fields in cerebral blood vessel data sets. Through testing on simulated images, phantom data, and cerebrovascular 4D flow data from 20 patients, the trained network successfully de-noised flow images, decreased velocity error, and enhanced near-vessel-wall velocity quantification and visualization. Such image enhancement can improve experimental and clinical qualitative and quantitative cerebrovascular PC MRI analysis.

## Introduction

Hemodynamic metrics can be of great value in cerebrovascular disease diagnosis and treatment planning^1–7^. Such metrics can be derived or calculated with a variety of medical imaging and computational methods. One method that has emerged as a powerful, in-vivo technique is the direct measurement of blood velocities with four-dimensional flow (4D-flow) magnetic resonance imaging (MRI), which is a three-dimensional, time-resolved form of phase contrast (PC) MRI^8,9^. 4D-flow can be used to non-invasively visualize and quantify blood flow in complex cerebrovascular systems; however, flow analysis with MRI has some limitations when used as a stand-alone analysis method. For example, flow resolution limits can result from patient scan time restrictions and from imperfections that result from manipulation of a magnetic field (image noise, intravoxel dephasing, eddy currents, magnetic field non-linearity, etc.). Another widely available cerebral blood flow analysis method, which may help address some of these limitations, is computational fluid dynamics (CFD)^10–12^. CFD is a method that utilizes the governing equations of fluid flow to calculate a velocity field from input geometric data and flow conditions. Because CFD is computationally based, there is almost no limit to its flow resolution, given the appropriate computational resources. Furthermore, computational model conditions can be manipulated to match physiological or surgical variations of interest. However, standalone CFD can also be limited due to its high dependence on patient-specific input conditions (boundary conditions), and its need for appropriate verification and validation. Therefore, a method that utilizes the best of both MRI and CFD may allow for enhanced cerebrovascular flow analysis by leveraging the advantages of one method to fill the inherent gaps of the other.

A number of studies have been aimed at taking advantage of the synergistic strengths of PC MRI and computational simulation to address current flow characterization limitations in a variety of vascular territories. For example, computer simulations have been used to create divergence free velocity fields from PC MRI, to analyze the effect of 4D flow MRI velocity resolution on viscous dissipation quantification, to assess the accuracy of MRI wall shear stress, and to improve PC MRI velocity resolution and noise performance with innovative CFD-based reconstruction and velocity-field optimization techniques^13–17^. Many studies have also used PC MRI data to improve computational simulation relevance by integrating PC MRI data as boundary conditions and even regularizing CFD by 3D PC MRI^18–22^. Furthermore, data from CFD and PC MRI are often compared to move towards validation of quantitative and qualitative flow characterization^23^. Building on these advancements, a more robust flow physics-based method that allows for retrospective correction of PC MRI could prove highly beneficial for experimental and clinical cardiovascular flow analysis with MRI. However, the large data sets produced by each of these time-resolved and three-dimensional methods, and the number of data sets required to create an extensive knowledge base of patient-specific flow regimes, have made the development of such a method challenging.

In addition to recent developments in medical imaging, machine learning has played an integral role in the advancement of cardiovascular analysis^24^. Machine learning, and related neural network methods, can be used to learn input features of data and then use the features to generate useful output. Application of machine learning to cardiovascular MRI has led to a number of advancements, including improved image segmentation, reading and interpreting ECG tracings, improved image characterization, faster anatomical and pathological feature detection and measurement, and quicker cardiovascular disease prognosis and treatment^24–29^. Physics-informed machine learning methods have also recently been applied to improve cardiovascular pressure predictions^30^. More recently, machine learning techniques have been developed to improve the resolution of 4D-flow estimates in the aorta using CFD from three aortic geometries^31^. Yet, application of machine learning methods to improve the velocity fields of phase contrast MRI has been limited outside of these recent studies, despite its potential for cerebrovascular flow quantification improvement. Therefore, the purpose of this work was to develop a robust machine learning paradigm which fuses information from 4D flow MRI and CFD using supervised learning, to provide high resolution, physics-based, patient-specific flow fields in cerebrovascular anatomy.

## Methods

**Figure 1:**
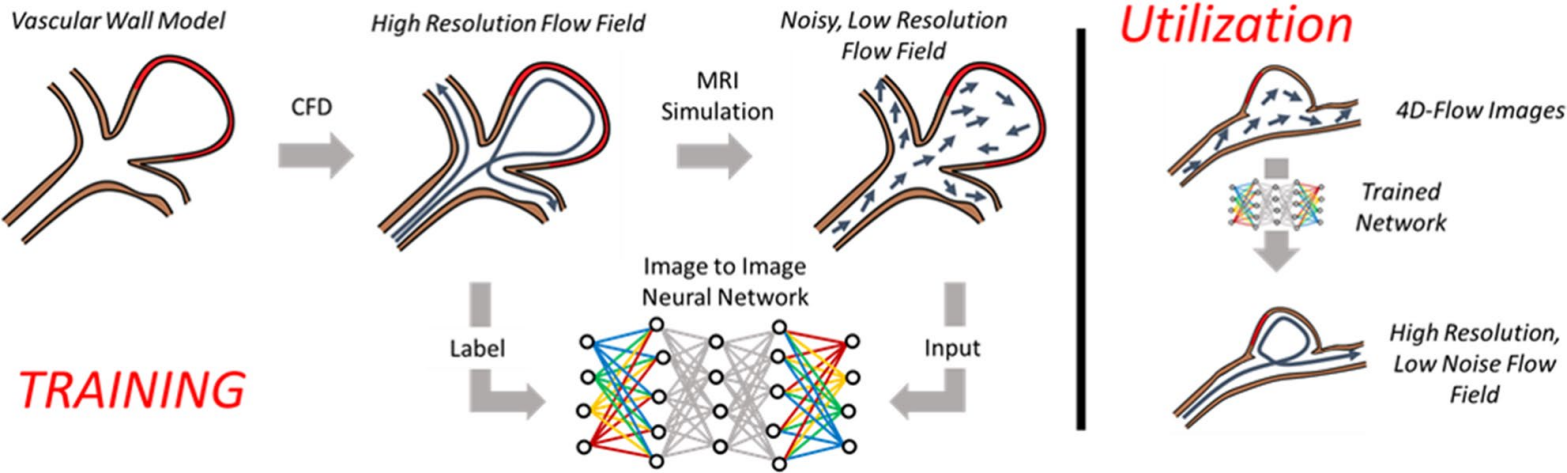
Method overview. Computational fluid dynamics (CFD) data was used to train a neural network. Network output was then applied to estimate high resolution, low noise flow fields from standard 4D flow MRI data.

The following sections detail the process of fusing CFD and 4D flow MRI data with supervised neural network training. An overview of this process is depicted in Fig. 1. This HIPAA-compliant study was approved by the University of Wisconsin-Madison, Health Science Institutional Review Board. All human subject data was obtained with informed written consent.

### Computational model generation

**Figure 2:**
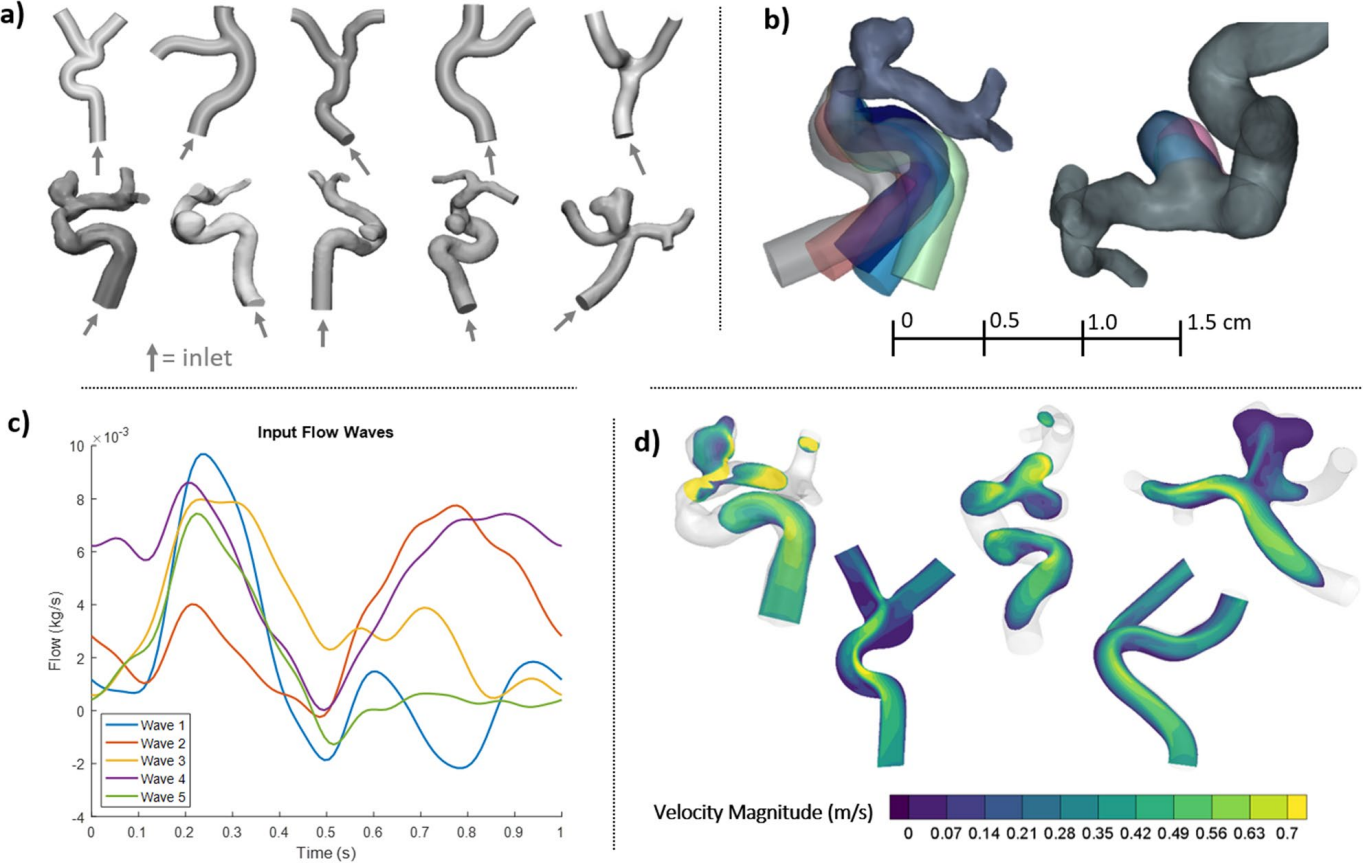
Computational fluid dynamics (CFD) simulations were performed on (**a**) 5 idealized blood vessel models, 5 patient-specific blood vessel models, and (**b**) 20 additional models that were manually modified from patient-derived models. (**c**) Six sets of unique inflow conditions were used with each geometry to create 180 total CFD simulations. (**d**) Example CFD results from representative models.

To generate a large number of realistic velocity field data sets on which a neural network could be trained, computational fluid dynamics simulations were prepared on vascular flow models. Five patient-specific cerebral aneurysm vessel flow path geometries were obtained from a data bank of de-identified high resolution digital-subtraction angiography images (Fig. 2a). These geometries were then virtually cut normal to the vessel flow path to make inlet and outlet boundaries, and smoothed to remove any artifact from the walls. To increase the amount of available flow geometries for CFD simulations, each of the original 5 patient-specific models was manually modified to create 5 additional iterations each. This resulted in 25 flow paths derived from the patient-specific data. Manual modification of these models involved alterations of vessel inlet angle, diameter increase or decrease, and change of aneurysm size and shape (Fig. 2b) in 3-matic (Materialise, Leuven, Belgium). Finally, to introduce additional variability in vessel flow path type, 5 fully synthetic flow path models were designed in Solidworks (Dessault Systemes). As shown in Fig. 2a, these idealized geometries did not include aneurysm sacs. In total, 30 vessel geometries were prepared for CFD simulation.

### Computational fluid dynamics simulations

The 30 vascular flow path geometries were individually imported into CONVERGE Studio (Convergent Science, Madison, WI) to set up CFD cases. To do this, a computational hexahedral grid was first created for each geometry model, with cut-cells used at vessel walls to accommodate the curved surfaces. A grid refinement study was performed to determine an appropriate grid cell size to achieve a grid-independent solution. This resulted in an approximate cell side length to vessel diameter ratio of less than 0.05. Rigid walls were assumed for these simulations, and no-slip conditions were imposed at the boundaries. A time-varying flow profile was set at the inlet boundary, and pressure values were set at each outlet. Blood properties were input for the simulation working fluid (density = 1060 kg/m^3^; viscosity = 0.004 Ns/m^2^). Reynold numbers ranged from about 100–800 across various geometric location and time-varying flow profiles. Finite volume simulations were run at a constant grid (no adaptive mesh refinement), and at a variable time step (the time step adapted with the simulation solution). A pressure implicit with splitting of operators (PISO) algorithm was used to solve the governing equations of fluid flow in CONVERGE CFD software (Convergent Science, Madison, WI). Rhie-Chow interpolation was used to maintain collocated variables, and a monotonic upstream-centered scheme for conservation laws (MUSCL) was used to provide second order spatial accuracy for convection terms. Time steps were limited by diffusive and convective CFL parameters. Solution results were exported every 0.02 s of simulated time. Simulations were repeated six times for each geometry, with six different inlet flow profiles, as displayed in Fig. 2c. This resulted in a total of 180 unique time-varying velocity fields to be used for neural network training. Each simulation took between 2 and 12 h, depending on availability of resources at a local high throughput computing center. Example CFD flow field results are displayed in Fig. 2d.

### Data preparation

CFD velocity data was processed to create pairs consisting of high-resolution flow fields with and without corruption, representing errors introduced from the MRI measurement process. To do this, CFD data was first converted to a uniform Cartesian grid. This conversion placed out-flow and in-flow boundaries into free-space, resulting in unrealistic flow fields in these areas. Unrealistic boundary edge effects were accounted for during training, as described below. Cut-cells from the CFD grid were also accounted for in the data conversion by weighting the velocity values of each cell by the cell volume. The application of this fix ensured realistic velocities at the edge of the CFD-based MR simulated data.

After the high-resolution data had been converted to a Cartesian representation, simulated MRI data was generated for use in network training. MR images were simulated using a model that accounted for complex valued operations and data corruption from k-space sampling, noise, signal magnitude variations, and static background tissue. All MRI simulations were performed on full Cartesian volumes, prior to any block operations performed for training. Simulations were performed in real-time during training, with a new simulation performed every time a cased was used. First, the velocity field was rotated in 3D space using a random rotation and cubic-spline interpolations. A synthetic background magnitude signal was then created by randomly generating complex k-space magnitude over a 5 × 5 × 5 grid using a standard normal distribution. These grids were Fourier transformed to image space and soft-thresholded to create spatially varying background signal with high resolution edges corresponding to tissue boundaries. Finally, this background signal was additively combined with vessel mask, with the background to vessel ratio ranging from 0 to 2. Next, the complex signal was created from the background magnitude signal and the velocity data using the standard PC-MRI model with a randomly selected V_enc_ from 0.1 to 0.9 of the peak velocity. Finally, the complex data was Fourier transformed, corrupted with additive complex, white Gaussian noise corresponding to SNR ranging from 10 to 10,000, cropped to 0.25 its original spatial resolution, and Fourier transformed back.

### Network and training

The simulated flow fields were used to train a convolutional neural network (CNN) (see supplemental Fig. 1) {Medero, 2020, In Vitro Assessment of Flow Variability in an Intracranial Aneurysm Model Using 4D Flow MRI and Tomographic PIV}. Architecture and models were based on a block-wise residual architecture similar to those used in state-of-the-art super resolution approaches^32–42^. After simulating the MRI acquisition, 32 × 32 × 32 blocks were randomly extracted from the velocity field. To identify edges and segments with artifacts due to resampling, segments of data were checked for mass conservation by summing the flows in and out of a designated block of data. If the block had a non-zero source or sink, then it was not used and a new randomly selected block was selected. Through this process, any edges that did not satisfy continuity (conservation of mass) due to partial volumes of inlets and outlets in the CFD data were eliminated. Training was performed with a batch size of 8, with 32 randomly selected blocks used before a new MRI simulation was performed. On a block by block basis, augmentation was performed using random flips of the velocity image and its velocity. Training used a magnitude weighted least squares loss function to account for the inverse relationship between magnitude and velocity noise. All processing was performed in python with PyTorch for machine learning operations (Open Source, https://github.com/pytorch/pytorch) and SigPy for data augmentations (Open Source, https://github.com/mikgroup/sigpy). Training was performed on a Titan RTX. An 80/20 split was used for training and validation with testing performed on newly acquired data not involved in the training.

### Evaluation

#### In-vitro phantom test case with high resolution 4D flow MRI

To evaluate the network, high resolution 4D flow MR imaging was performed on a physical, patient specific blood vessel phantom (see supplemental Fig. 2). First, a cerebral vasculature model was created by 3D-printing a dissolvable vessel core, casting the core in optically clear silicone, and then dissolving the vessel core from the cured silicone block, as described in past work^43^. The model was integrated into a cardiovascular flow loop in line with a pulsatile flow system (PD-1100, BDC Laboratory, Wheat Ridge, CO). The model was then placed on the bed of a 3 T MR imaging system (Signa Premier, GE Healthcare, Waukesha, WI), where high resolution 4D flow MRI data was acquired with PC-VIPR^9^. Imaging parameters included a 0.41 mm isotropic spatial resolution, 256 mm field of view, 9 ms repetition time, 100 cm/s velocity encoding, and 24-min scan time. The 4D flow data were reconstructed into 20 time frames per flow pump cycle. Phase offsets for Maxwell terms and eddy currents were corrected automatically during reconstruction. The eddy current correction was performed using 2nd order polynomial fitting of background tissue segmented based on thresholding of an angiogram. Velocity-weighted angiograms were calculated from the final velocity and magnitude data for all 20 time frames. The 24-min scan data was also sub-sampled into 12- and 6-min acquisitions, and reconstructed for each subsampling. The trained CNN was applied to the 6-, 12-, and 24-min velocity fields. The post-trained 6- and 12- minute velocity fields were compared to the 24-min scan as the high resolution standard. Comparisons were also made across all post-trained phantom scans (6- through 24-min). Quantitative analyses were performed in ImageJ (National Institutes for Health) and Matlab (Mathworks, Natick, MA), and qualitative analysis was performed in Ensight (CEI, Apex, NC). Images from each pre- and post- CNN enhanced data set were compared using a root-mean-square error metric (RMSE) as follows:

$$RMSE = \sqrt {\frac{{\sum\nolimits_{i = 1}^{n} {\left( {\hat{v}_{i} - v_{i} } \right)^{2} } }}{n}}$$

where $\hat{v}_{i}$ is the observed velocity metric of the first image, $v_{i}$ is the velocity metric of the comparison image, and n is the number of data points.

#### Subjective Evaluation on Subject-Specific 4D Flow MRI Data

To provide in-vivo evaluation, the network performance was evaluated in cerebral time-averaged 4D flow MRI data from 20 patients. Anonymized images were obtained from cerebral blood flow scans using PC-VIPR. Imaging parameters included a 0.6 mm isotropic spatial resolution, 100 cm/s velocity encoding, and 7 min and 32 s scan time. The trained network was applied to the reconstructed images, and noise and spatial resolution were compared between pre- and post- network application. PC-MR angiograms were also compared between the original subject data set and the post-applied network data set. To analyze flow quantification, vessel boundaries were manually segmented from each patient flow case, and flow metrics of both original (pre-trained) and enhanced (post-trained) data sets were measured in Ensight (CEI, Apex, NC, United States) and Matlab (Mathworks, Natick, MA). Flow metrics of interest included wall shear stress, blood flow kinetic energy, maximum velocity at cut planes in the internal carotid artery (ICA) and middle carotid artery (MCA), flow at planes in the ICA and MCA, vorticity at planes in the ICA and MCA, and the spatial distribution of velocity at ICA and MCA planes. Velocity vectors and streamlines were also plotted at select areas of the patient vasculature to facilitate qualitative comparisons. Quantitative statistical comparisons were made with a Student’s paired t-test (α = 0.05, two-tailed), linear correlation, and Bland Altman analysis after checking for approximate normality of the velocity-based data.

#### CFD “Test” case

Although the physiological accuracy of patient-specific CFD simulations can be limited by the accuracy of the boundary conditions, the true and estimated velocity fields generated by CFD can be well characterized because if its high resolution and basis in physics. Therefore, to analysis the difference between a CFD “truth” velocity field and the network “estimated” velocity field an additional CFD case was made and run through the network. For this CFD case, a new DSA-based vascular model (different than the geometries used for training) was segmented and set up for CFD, and a flow field was simulated using the same CFD setup parameters as used for the training data set. The flow field was converted to image format and run through the already-trained network. The velocity field mean square error was then compared with and without the CNN.

## Results

**Figure 3:**
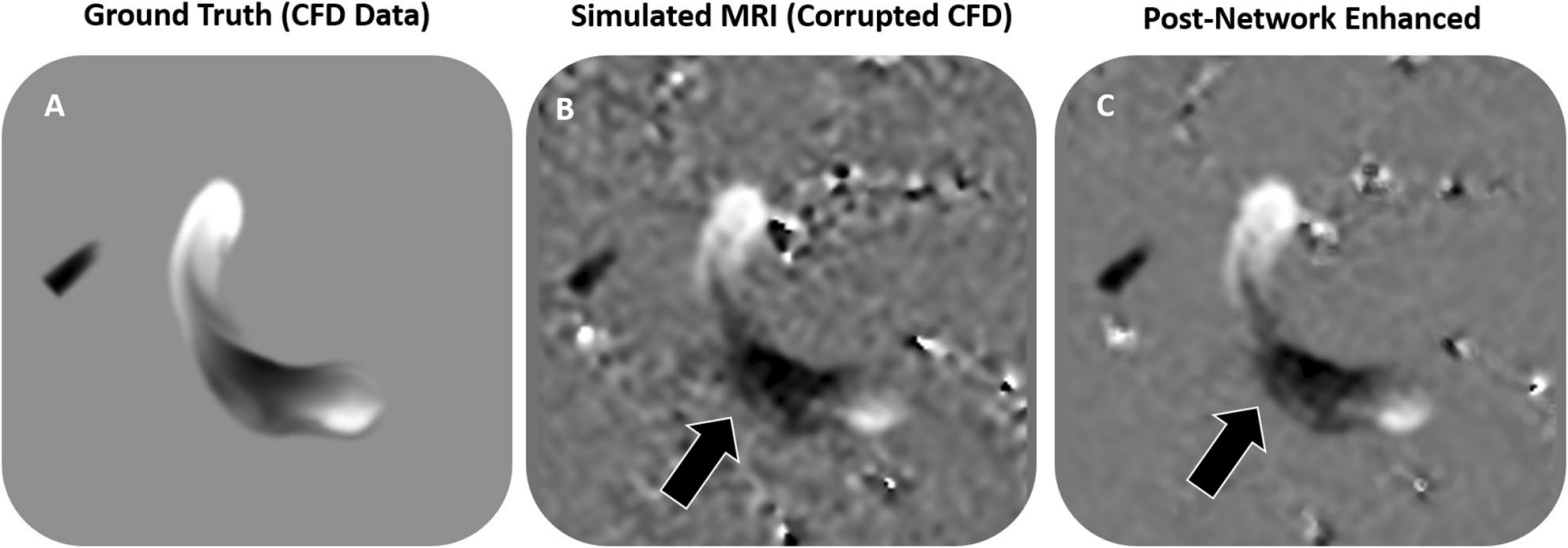
The trained neural network output de-noises manually corrupted data. In this example, (**a**) clean CFD data was (**b**) corrupted using a simulated MRI acquisition, and (**c**) enhanced with the trained neural network.

The network was trained over 5 days with no sign of overfitting, likely due to the aggressive augmentation. Validation and test case results are plotted in Supplemental Figs. 3 and 4. Application of the trained neural network on a CFD test-case data produced a significant reduction in image noise (Fig. 3). When the trained network was applied to a corrupted velocity field, background noise was decreased by 64%. Furthermore, velocity error was reduced from 20% in the corrupted velocity field to 8% in the post-training velocity field when the original CFD velocity field was taken as the ground truth.

**Figure 4:**
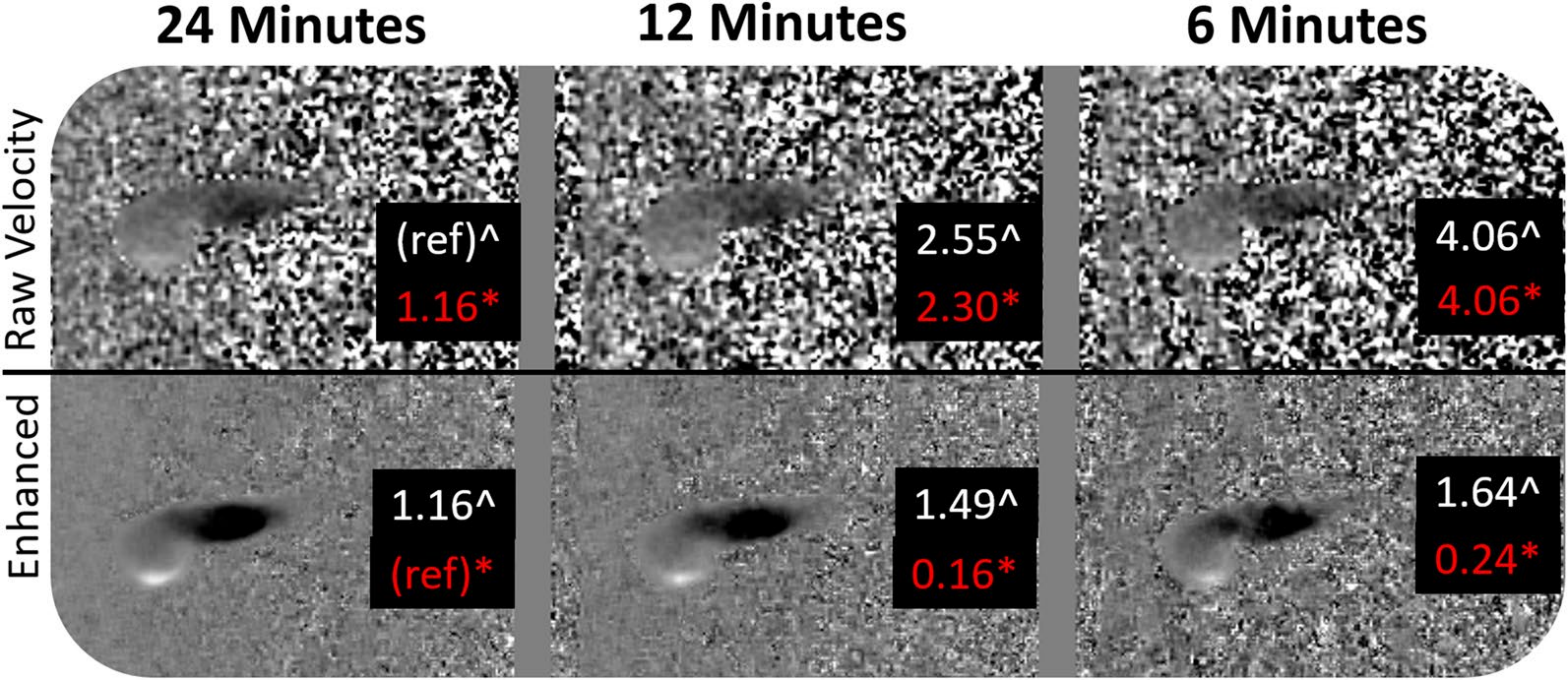
Physical phantom experiment output. A high resolution (24-min) 4D flow MRI procedure was performed on a blood vessel phantom. Subsequently, the data was sub-sampled into 12- and 6- minute scans. The trained neural network was applied to all three data sets (Enhanced). Data was first compared to the raw 24-min 4D flow scan data with a root-mean-square error (RMSE) metric (denoted by ^). The data was then compared to the “enhanced” data that was cleaned with the trained network (RMSE denoted by *).

Application of the trained network to the experimental physical phantom data also produced a significant reduction in background noise (Fig. 4). When the high resolution 24-min 4D flow MRI raw velocity was taken as the reference data set, RMSE was reduced from 2.55 to 1.49 in the 12-min sub-sample scan data, and from 4.06 to 1.64 in the 6-min sub-sample scan. When the enhanced (post-trained) 24-min scan was used as the reference, the enhanced 12-min and 6-min scan data sets produced a RMSE of 0.16 and 0.24, respectively. The time-varying flow fields of the original and enhanced phantom cases can be seen in supplemental video 1.

**Figure 5:**
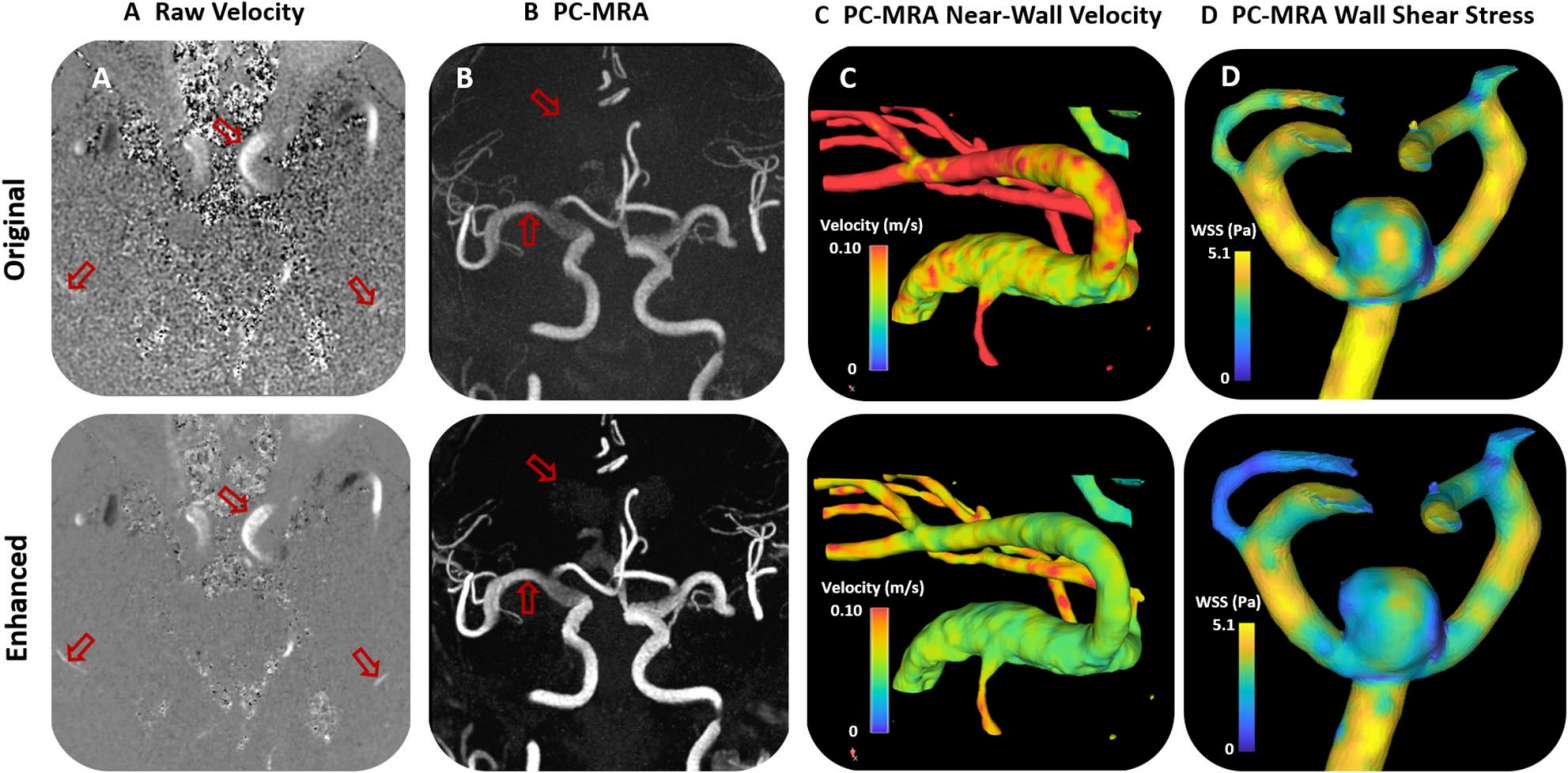
Patient cerebral flow MRI results. (**a**) Cerebral 4D flow MRI raw velocity images before and after trained network application. (**b**) Phase-contrast MR angiogram images before and after trained network application. (**c**) Near wall velocity contours displayed on cerebrovascular segmentations before and after network application. (**d**) Wall shear stress plots on a cerebral aneurysm wall. Arrows note key areas of image improvement.

**Figure 6:**
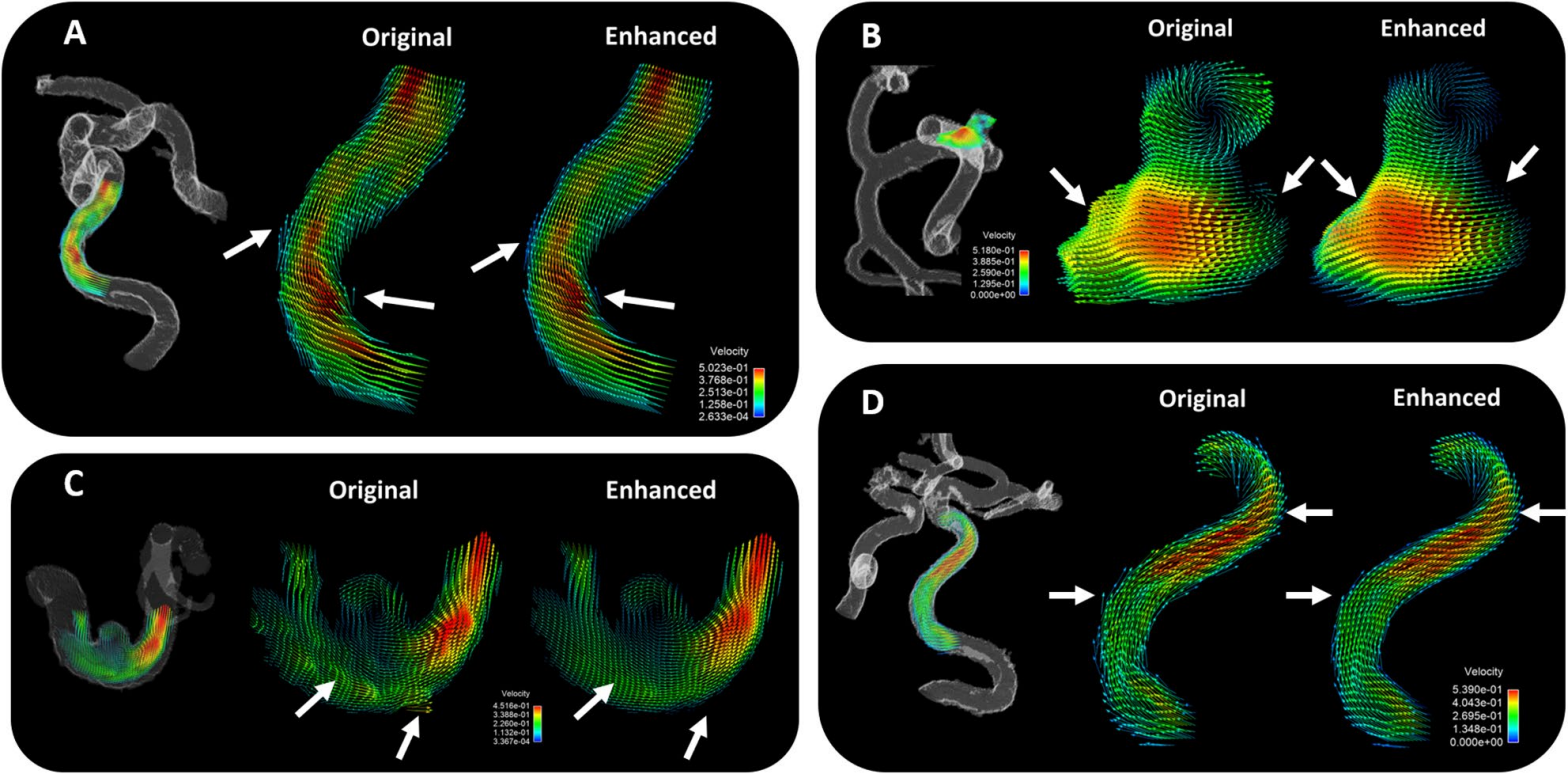
Velocity vectors in select planes of patient cerebrovascular anatomy. (**A**) Near wall vector alignment and velocity gradients are improved with ML enhancement, as displayed on a transverse plane through an internal carotid artery (ICA) (**B**) Smoother velocity gradient transitions in are observed in the ML enhanced data of a cerebral aneurysm and its surrounding vasculature structure (**C**) Unrealistic velocity fluctuations are removed during ML enhancement, as observed in the parent vessel surrounding a cerebral aneurysm (**D**) Near wall velocity characterization is cleaned in a tortuous ICA segment, which had a large impact on quantification of near-wall flow metrics, such as wall shear stress.

**Figure 7:**
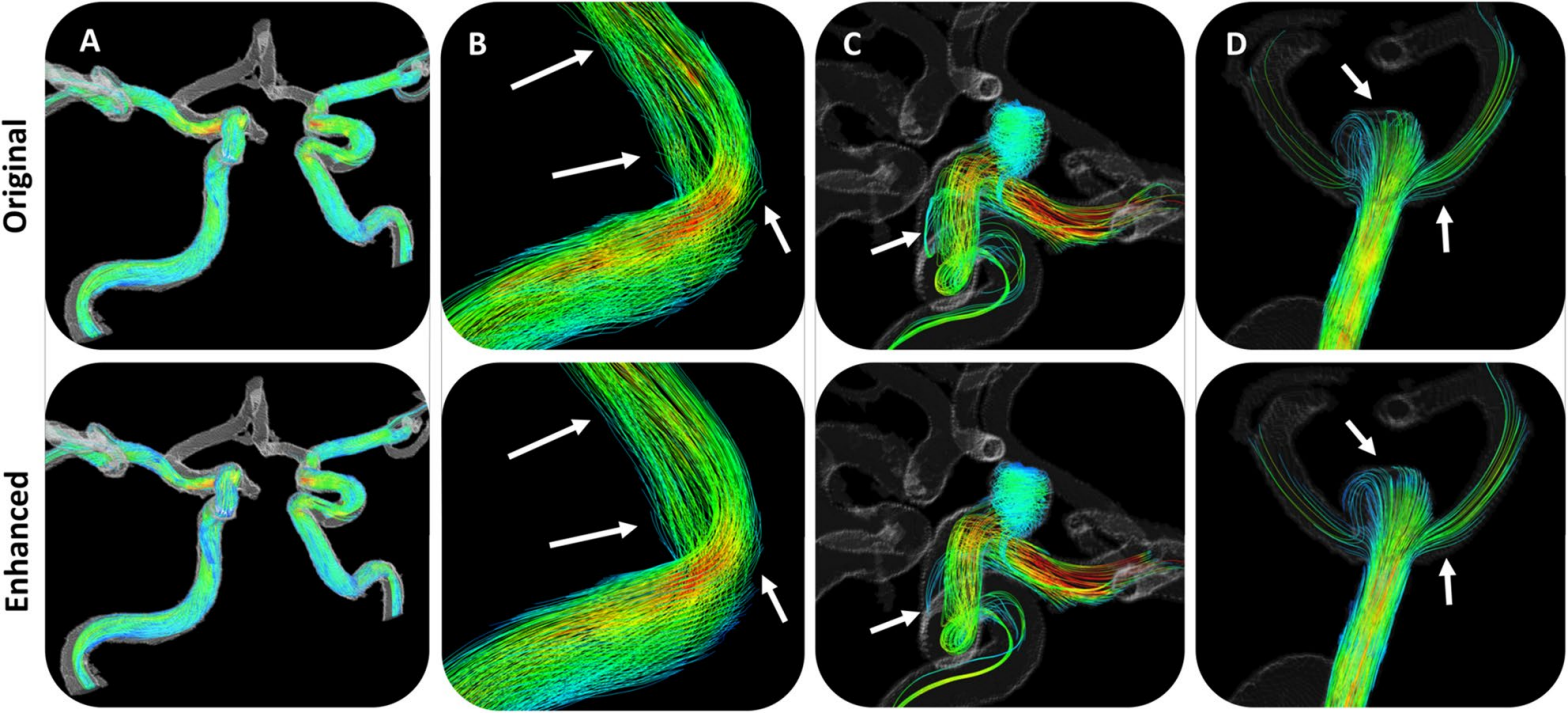
Velocity streamlines throughout select patient cerebrovascular anatomy. (**A**) A broad view of streamlines through the right and left ICA and MCA reveals no major difference in streamlines length or quality between original and enhanced flow fields, however a zoomed in view of an ICA vessel bend in (**B**) shows that streamlines from the original flow field tend to point and terminate outside of the vessel wall, while streamlines in the enhanced flow field tend to stay normal to the path of flow. (**C**) Streamlines in an ICA aneurysm (**D**) Streamlines in an MCA aneurysm.

**Figure 8:**
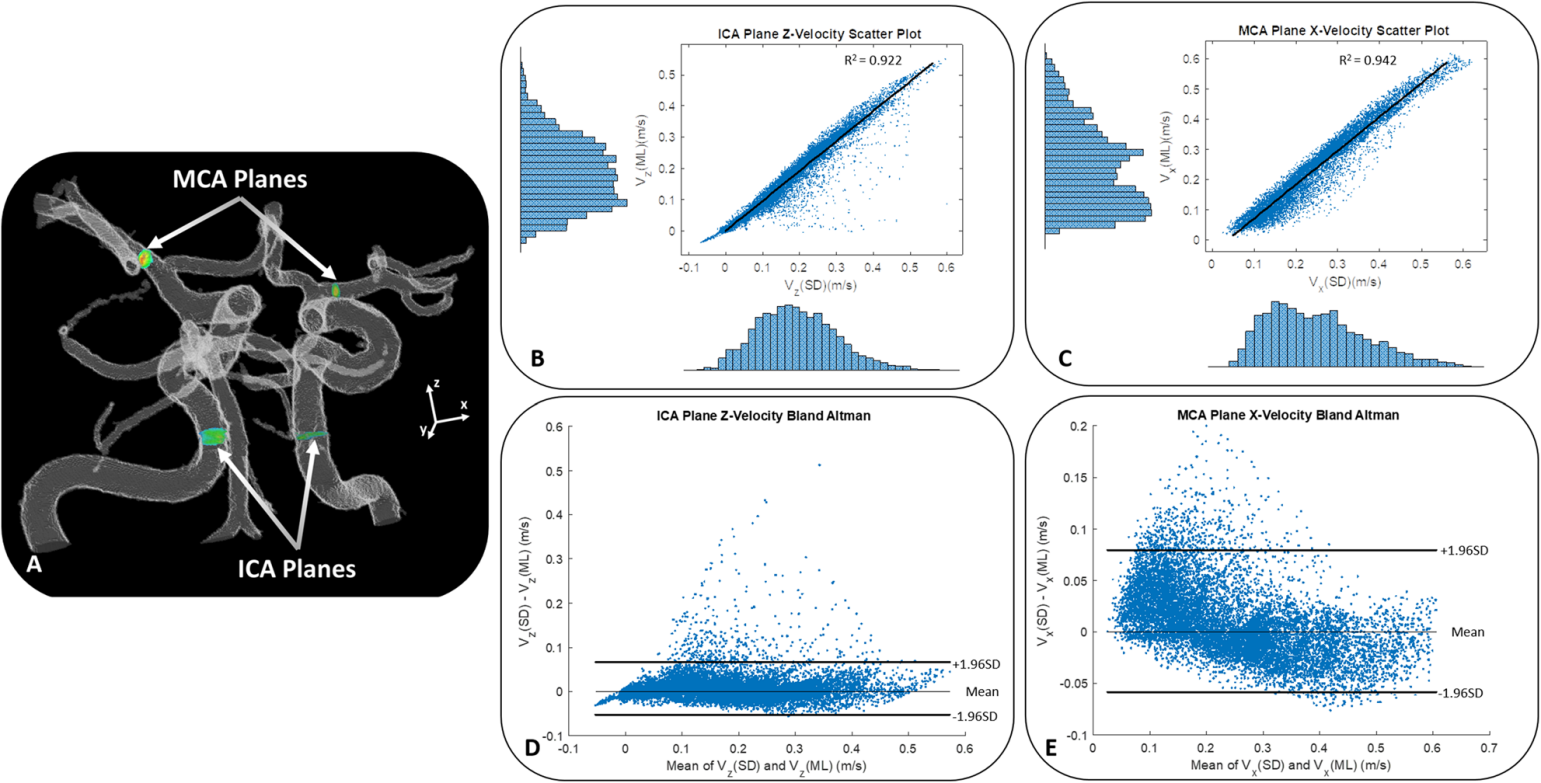
Velocity values from each voxel intersected by planes placed in the ICA and MCA were plotted for 20 pre- and post- enhanced patient cerebrovascular 4D flow MRI cases. (**A**) Representative ICA and MCA plane locations shown on a cerebrovascular mask. The original (SD for standard) and enhanced (ML for machine learned) 4D flow velocities from the 20 cases were correlated for both the (**B**) ICA z-direction velocities and the (**C**) MCA x-direction velocities. Bland Altman plots of these same velocity values were plotted for both the (**D**) ICA and (**E**) MCA planar data. Note that the data falling outside of the “normal” range represents near-wall velocity data, which was corrected through enhancement of the near wall velocity gradients through training. The near-wall velocity enhancements had a large impact on wall shear stress quantification.

As with the CFD test case and experimental model case, the application of the trained neural network improved image results in the 20 patient 4D flow MRI data sets. As shown in Fig. 5, the CNN-enhanced velocity images had lower noise and higher apparent spatial resolution than the raw velocity images. Additionally, as seen in Fig. 5, the CNN-enhanced PC-MRA had greater vessel boundary delineation. Accordingly, the velocity vector and streamline representation was also improved in CNN-cleaned images, particularly near the vessel walls (Figs. 5, 6, and 7). When the original (pre-trained) and enhanced (post-trained) ICA and MCA plane velocities were plotted vs each other (Fig. 8), a strong correlation could be observed. Velocity points that fell outside of the strong correlative trend tended to be located near the vessel walls, as these points were most affected by network enhancement. As shown in supplemental table 1, comparison of quantitative results revealed that the wall shear stress (Original = 3.15 ± 0.28 95%CI Pa; Enhanced = 2.25 ± 0.29 95%CI Pa; *p* < 0.001) and kinetic energy (Original = 1.29E−04 ± 1.96E−04 95%CI mJ; Enhanced = 1.18E−04 ± 1.96E−04 95%CI; *p* < 0.001) were significantly lower in the machine learning enhanced data than it was in the original pre-trained data. Maximum ICA velocity in both the left (*p* = 0.001) and right (*p* = 0.001) brain, ICA flow in the left brain (*p* = 0.018), and MCA flow in both the left (*p* < 0.001) and right (*p* = 0.029) brain were all significantly lower in the enhanced flow data than they were in the original data [data in supplemental table 1]. MCA vorticity in the left brain was significantly higher in the enhanced flow data (*p* = 0.003). There were no significant differences in the rest of the flow metrics that were analyzed.

## Discussion

Augmentation of 4D flow MRI data with CFD-informed training networks can produce enhanced physiological flow fields. In this preliminary work, the potential utility of such a method was demonstrated by using high resolution patient-specific CFD data to train a neural network, and then using the trained network to enhance MRI-derived cerebrovascular velocity fields. Through testing on simulated images, phantom data, and in-vivo human subject data, the trained network successfully de-noised flow images and decreased velocity error, particularly near vessel walls. Such image enhancement has the potential to significantly improve experimental and clinical qualitative and quantitative PC MRI analysis of cerebrovascular flow.

PC MRI has proven to be an immensely beneficial tool for cardiovascular flow analysis^8,21,44^. By providing a method to assess time-varying blood flow in two or three-dimensions, PC MRI provides valuable visualization of complex blood flow that would otherwise be unavailable to clinicians and researchers. However, when using PC MRI as an analytical tool, it is important to ensure that the results being produced are representative of real physics-based, and physiologically representative, fluid flow. Much work has been put into this pursuit, resulting in advancements in image resolution and image error reduction though data acquisition and reconstruction improvements. And although it is important to keep making these improvements in PC MRI methods, flow characterization with MRI may be improved through other complementary tools and techniques as well. In this work, it was shown that both machine learning and computational simulation techniques can be leveraged to improve PC MRI data. The utilization of CFD methods created a foundational physics-based data set to which a neural network could be trained, in addition to a high resolution standard to which lower resolution PC MRI data could be compared. Furthermore, the use of machine learning techniques allowed for the time-effective utilization of large and complex data sets.

In the training of the network, high resolution “ground truth” CFD data was used as a comparison standard for network output. Through this test, it was shown that manually corrupted velocity data could be largely corrected through application of a CFD-data-trained neural network. That is, the network can certainly learn to partially remove the corruption, although with substantial training time. And, although this test was limited in PC MRI applicability because it only entailed un-corrupting data that was previously manually corrupted, it provided a good check to how the network was adapting in the iterative phase of network development. Additionally, the high resolution CFD data provided a ground truth metric that is otherwise unavailable with in-vivo subject images.

In the first network test, involving high resolution 4D flow MRI on an anatomical-based phantom, the network was also successful in reducing image noise and decreasing velocity RMS error. In this case, the high resolution 24-min 4D flow MRI scan, which is prohibitively long for clinical use, provided a reference standard. Comparisons to this high resolution scan not only displayed the utility of image enhancement that the trained neural network provides, but also demonstrated the large potential for future error reduction over current clinically-used 4 to 12 min protocols. Such phantom studies will be continued for future development of this work, in addition to validation studies with gold-standard experimental techniques.

In addition to the development of PC MRI visualization for cardiovascular flow analysis, there have been many efforts to improve the quantitative capabilities of PC MRI. It is well known that errors in PC MRI velocity estimation can have a large influence on quantitative analysis metrics that are calculated through post-processing^14,45–47^. In particular, noise errors, limitations in velocity range acquisition (velocity encoding settings), and near-wall velocity error can negatively affect quantification of metrics such as flow energy dissipation, vorticity, flow distribution, stasis, pressure loss, and wall shear stress, among others^47–51^. Results of the final network test on the patient 4D flow MRI data demonstrated the potential utility of this proposed method in minimizing these errors. Through application of the trained network on cerebral flow images, a significant reduction in noise and apparent increase in spatial flow resolution were observed. Perhaps more importantly, velocity vector analysis revealed enhancement of near wall velocities, which influenced metrics that are based on near-wall velocity gradients, such as wall shear stress. In nearly every case, the post-enhancement velocity fields had lower near-wall velocities than the original, non-enhanced case. This is consistent with a previous experimental study that suggests standard 4D flow MRI may overestimate velocities in certain areas of cerebral vascular anatomy, such as in aneurysms^52^. A careful review of the qualitative results of this study also supports this notion. A review of the velocity vector plots reveals that the original (pre-enhancement) data sets displayed areas of abrupt velocity transition and velocity “hotspots” near the vessel boundary. In contrast, the ML-enhanced data sets display a more physically realistic velocity gradient that approaches zero near the vessel boundary. Velocity profile improvement can be further observed in the quantitative plots of enhanced planar velocities vs original planar velocities. Note that data points that fell out of the “normal” range on correlation and Bland Altman plots were often representative of near-wall velocities. Correction of these points in particular had a large influence on wall shear stress quantification. Such quantification can be particularly important for analysis and treatment planning of pathological cerebral vasculature, such as in the case of cerebral aneurysms. Of additional importance, vorticity and kinetic energy metrics were observed to change in post-trained data. We presume that the bulk of vorticity and kinetic energy change was a result of the lower noise levels and improved near-wall velocity profiles in post-trained images. Lastly, as future work will explore, the CFD-trained network is independent of velocity encoding influence, and therefore may be used to correct areas of low or high velocity that were not fully characterized with in-vivo imaging.

There were a number of limitations in this preliminary study that warrant discussion. First of all, there are many PC MRI errors that were not considered in this work, such as aliasing and phase wrapping, intra-voxel dephasing, image artifacts, resolution limits, and more. It is acknowledged that these could have an influence on error in PC MRI, and they will be further investigated, in terms of their relation to the trained CNN, in future work. Second, the CFD training data set was intended to be preliminary, and therefore is somewhat limited. The data set used in this study was based on small cerebral vessels, with input conditions representative of cerebral flow. Additionally, the data set as a whole (180 simulations) is small relative to typical machine learning standards. Future work will expand the training data sets by introducing a wider variety of vessel types and flow regimes. Lastly, the human subject data application was also limited due to the preliminary nature of this work. Note, however, that ground truth CFD data and phantom data tend to work better with network testing and iteration, as we currently have no way of verifying which in-vivo velocity field (pre- or post-enhancement) is closer to the true velocity field. Accordingly, the network was tested on only twenty human data sets, which had relatively high image quality before enhancement. Future work will expand this human in-vivo data application with the intention of improving the quality of clinically-relevant flow results that can be obtained with 4D flow MRI.

## Supplementary information

Supplementary Information 1.

Supplementary Video 1.
