# Supplementary material for: Enhancement of cerebrovascular 4D flow MRI velocity fields using machine learning and computational fluid dynamics simulation data
Source: Sci Rep. 2021 May 13;11:10240. doi: 10.1038/s41598-021-89636-z (PMC8119419; doi:10.1038/s41598-021-89636-z)
Supplement: Supplementary file 1 — Supplementary Information 1. [file 41598_2021_89636_MOESM1_ESM.docx]

**Supplemental Material**

**Enhancement of cerebrovascular 4D flow MRI velocity fields using machine learning and computational fluid dynamics simulation data**

David Rutkowski^1,2^, Alejandro Roldán-Alzate^1,2^, Kevin M. Johnson^2,3^

^1^Mechanical Engineering, ^2^Radiology, ^3^Medical Physics, University of Wisconsin, Madison, WI USA

**Supplemental Video 1.** Time varying velocity images from flow phantom experiments.

**Supplemental Table 1.** Quantitative comparisons of velocity-based metrics derived from original and ML-enhanced 4D flow MRI data in 20 patient data sets. Analyzed using custom scripts in Ensight 10.0 (CEI, Inc)

|  |  | **Left Brain** | | | | | |
| --- | --- | --- | --- | --- | --- | --- | --- |
| *[±95%CI]* | *Mean WSS (Pa)* | *ICA Max Velocity (m/s)* | *ICA Flow (ml/min)* | *ICA Vorticity (1/s)* | *MCA Max Velocity (m/s)* | *MCA Flow (ml/min)* | *MCA Vorticity (1/s)* |
| Original | 3.15 *[0.28]* | 0.43 *[0.04]* | 196.2 *[23.2]* | 0.52 *[0.11]* | 0.49 *[0.05]* | 111.6 *[8.4]* | 0.71 *[0.12]* |
| Enhanced | 2.25 *[0.29]* | 0.38 *[0.04]* | 191.4 *[22.3]* | 0.46 *[0.06]* | 0.49 *[0.06]* | 108.6 *[8.2]* | 0.79 *[0.11]* |
| p-value | **<0.001** | **0.001** | **0.018** | 0.251 | 0.414 | **<0.001** | **0.003** |
|  |  | **Right Brain** | | | | | |
|  | *Mean KE (mJ)* | *ICA Max Velocity (m/s)* | *ICA Flow (ml/min)* | *ICA Vorticity (1/s)* | *MCA Max Velocity (m/s)* | *MCA Flow (ml/min)* | *MCA Vorticity (1/s)* |
| Original | 1.29E-04 *[1.96E-05]* | 0.41 *[0.04]* | 181.2 *[25.0]* | 0.48 *[0.07]* | 0.50 *[0.05]* | 107.4 *[9.4]* | 0.81 *[0.17]* |
| Enhanced | 1.18E-04 *[1.96E-05]* | 0.36 *[0.04]* | 183 *[21.3]* | 0.45*[0.06]* | 0.49 *[0.06]* | 102 *[11.1]* | 0.81 *[0.12]* |
| p-value | **<0.001** | **0.001** | 0.773 | 0.354 | 0.369 | **0.029** | 0.969 |


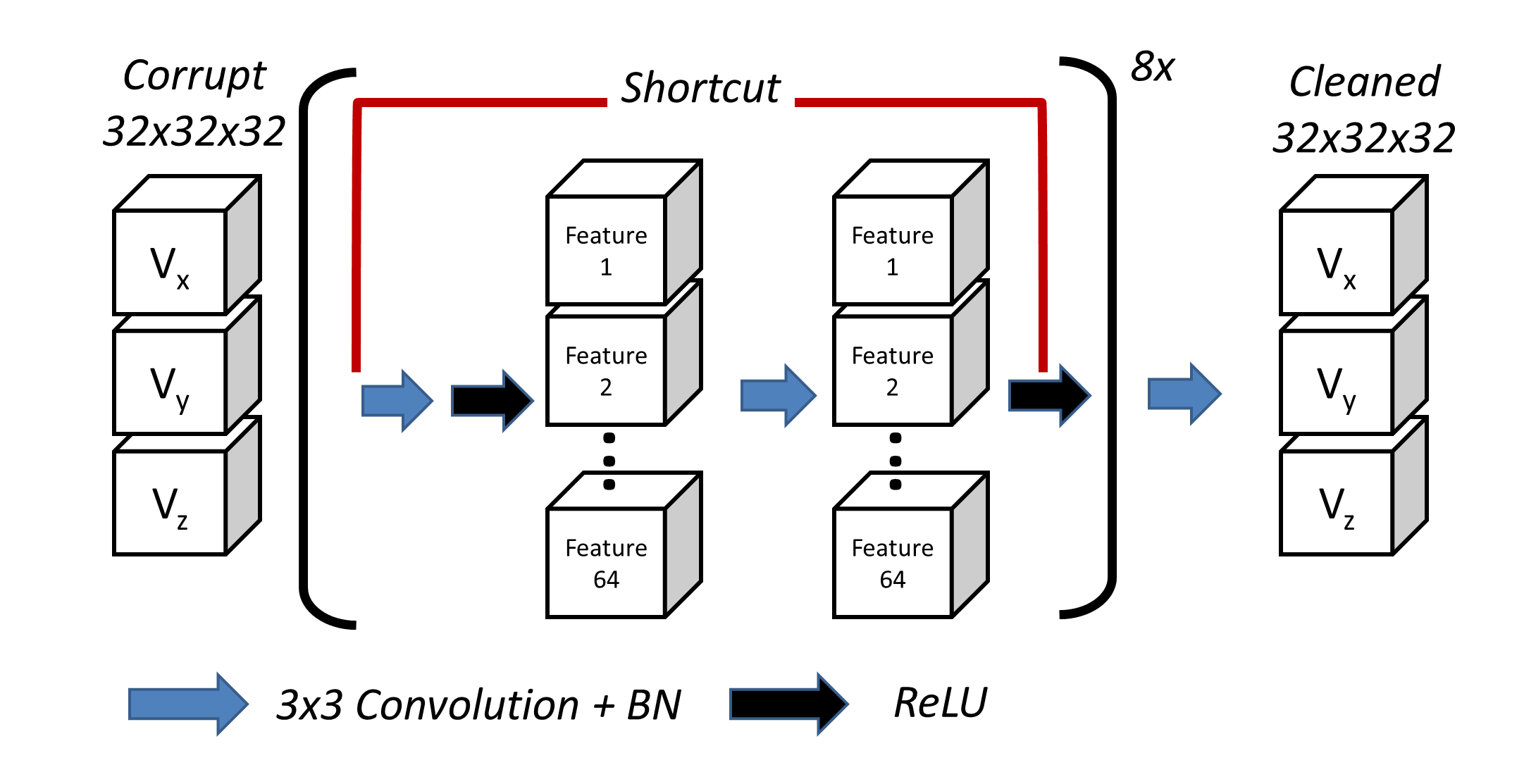


**Supplemental Figure 1.** Overview of the block-based neural network architecture and training workflow.


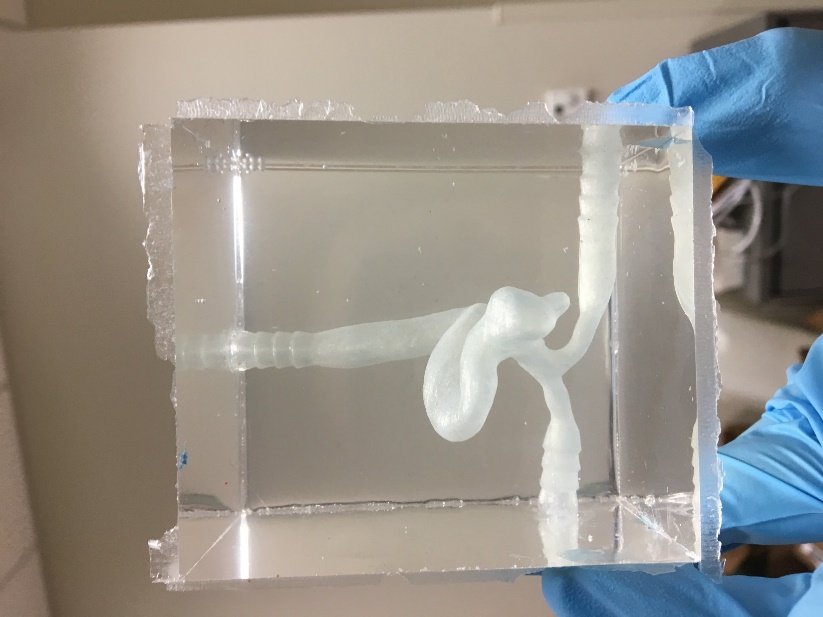


**Supplemental Figure 2.** An aneurysm flow phantom used for experimental testing of the network. This model was made by a silicone casting method. It was then integrated into a mock circulatory loop and placed on the MRI bed for high resolution 4D flow MRI acquisition.


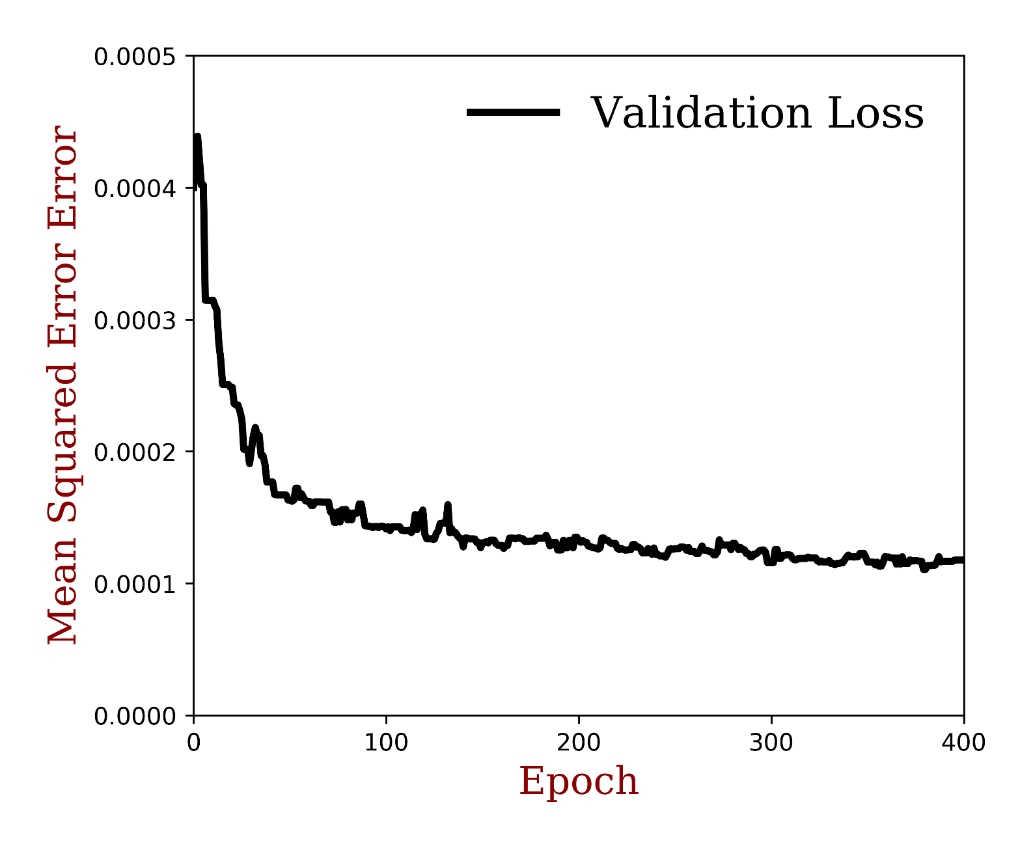


**Supplemental Figure 3.** Validation loss is plotted to show mean squared error reduction during training. Plotted with Matlab (Mathworks)


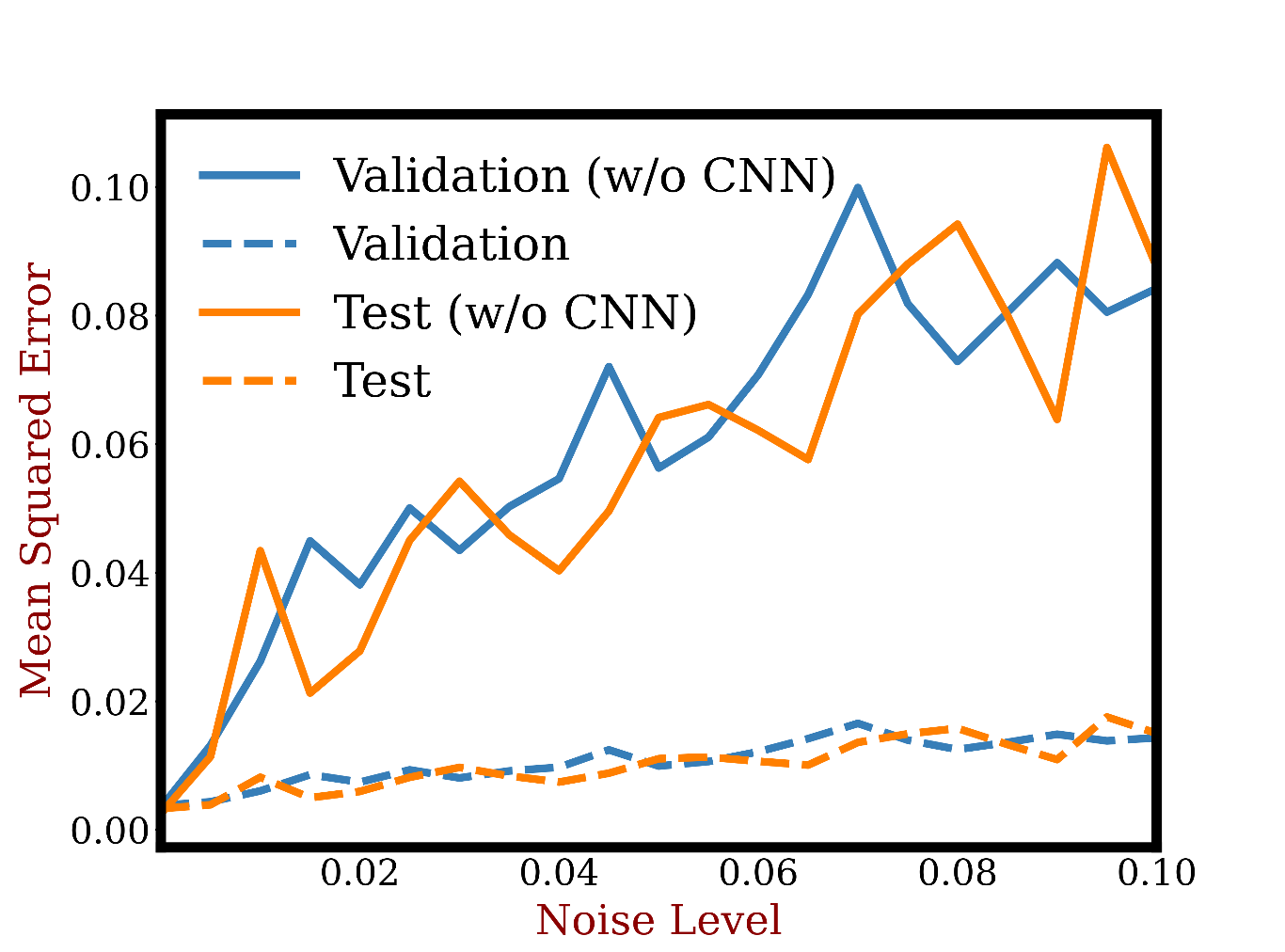


**Supplemental Figure 4.** A network validation was performed by running an additional CFD case, which was separate than those used for network training. Mean square error was plotted vs noise level for test and validation cases with and without the CNN. Plotted with Matlab (Mathworks)
